# Toxicokinetics and Toxicodynamics of Ayahuasca Alkaloids *N*,*N*-Dimethyltryptamine (DMT), Harmine, Harmaline and Tetrahydroharmine: Clinical and Forensic Impact

**DOI:** 10.3390/ph13110334

**Published:** 2020-10-23

**Authors:** Andreia Machado Brito-da-Costa, Diana Dias-da-Silva, Nelson G. M. Gomes, Ricardo Jorge Dinis-Oliveira, Áurea Madureira-Carvalho

**Affiliations:** 1 Department of Sciences, IINFACTS-Institute of Research and Advanced Training in Health Sciences and Technologies, University Institute of Health Sciences (IUCS), CESPU, CRL, 4585-116 Gandra, Portugal; a26127@alunos.cespu.pt (A.M.B.-d.-C.); ngomes@ff.up.pt (N.G.M.G.); aurea.carvalho@iucs.cespu.pt (Á.M.-C.); 2 UCIBIO-REQUIMTE, Laboratory of Toxicology, Department of Biological Sciences, Faculty of Pharmacy, University of Porto, 4050-313 Porto, Portugal; 3 LAQV-REQUIMTE, Laboratory of Pharmacognosy, Department of Chemistry, Faculty of Pharmacy, University of Porto, 4050-313 Porto, Portugal; 4 Department of Public Health and Forensic Sciences, and Medical Education, Faculty of Medicine, University of Porto, 4200-319 Porto, Portugal

**Keywords:** hallucinogens, metabolism, phytotherapy, recreational drugs

## Abstract

Ayahuasca is a hallucinogenic botanical beverage originally used by indigenous Amazonian tribes in religious ceremonies and therapeutic practices. While ethnobotanical surveys still indicate its spiritual and medicinal uses, consumption of ayahuasca has been progressively related with a recreational purpose, particularly in Western societies. The ayahuasca aqueous concoction is typically prepared from the leaves of the *N*,*N*-dimethyltryptamine (DMT)-containing *Psychotria viridis*, and the stem and bark of *Banisteriopsis caapi*, the plant source of harmala alkaloids. Herein, the toxicokinetics and toxicodynamics of the psychoactive DMT and harmala alkaloids harmine, harmaline and tetrahydroharmine, are comprehensively covered, particularly emphasizing the psychological, physiological, and toxic effects deriving from their concomitant intake. Potential therapeutic utility, particularly in mental and psychiatric disorders, and forensic aspects of DMT and ayahuasca are also reviewed and discussed. Following administration of ayahuasca, DMT is rapidly absorbed and distributed. Harmala alkaloids act as potent inhibitors of monoamine oxidase A (MAO-A), preventing extensive first-pass degradation of DMT into 3-indole-acetic acid (3-IAA), and enabling sufficient amounts of DMT to reach the brain. DMT has affinity for a variety of serotonergic and non-serotonergic receptors, though its psychotropic effects are mainly related with the activation of serotonin receptors type 2A (5-HT_2A_). Mildly to rarely severe psychedelic adverse effects are reported for ayahuasca or its alkaloids individually, but abuse does not lead to dependence or tolerance. For a long time, the evidence has pointed to potential psychotherapeutic benefits in the treatment of depression, anxiety, and substance abuse disorders; and although misuse of ayahuasca has been diverting attention away from such clinical potential, research onto its therapeutic effects has now strongly resurged.

## 1. Introduction

Naturally-occurring hallucinogens have been used for millennia by many indigenous cultures, not only in religious and spiritual ceremonies but also due to their medicinal benefits [1,2]. Their use in modern cultures witnessed a rapid spread in the 1960s and 1970s, mainly due to their popularity as recreational drugs [3]. These compounds are characterised by an overall capacity to induce mood alterations, variations in a person’s perception, mainly visual, and altered thought with overwhelming intellectual or spiritual insight, similar to what is experienced only through dreams or at times of religious exaltation [4,5]. In addition to the term *hallucinogen* (meaning that the drug produces hallucinations, while devoid of psychedelic effects at typical low doses) [1], other designations such as *entheogen*, *mysticomimetic*, or *psychotogen* were also used to label these compounds [2]. Ultimately, the most commonly used designation to describe the nature of these drugs is *psychedelic* (“mind revealing” or “mind-manifesting”), a term that do not focus on one specific effect [1]. Long-time classified as dangerous drugs by the media and regulatory agencies worldwide, psychedelics are very frequently devoid of serious toxic effects [3], psychoactivity being produced at low doses, usually insufficient to induce toxicity in a mammalian organism [1,6]. Additionally, their use does not lead to dependence, nor they are consumed for long periods of time [7,8], with unusual reports of chronic use [9].

Classical hallucinogens, also known as serotonergic substances, can be classified into two main chemical families: (i) phenylalkylamines, such as the well-known mescaline obtained from the peyote cactus (*Lophophora williamsii*) [10]; and (ii) indolylalkylamines that can be further divided into ergolines, like the semisynthetic lysergic acid diethylamide (LSD), and the simple tryptamines, including *N*,*N*-dimethyltryptamine (DMT) and the phosphorylated counterpart 4-phosphoryloxy-*N*,*N*-DMT (psilocybin) [4]. These psychedelics share a similar mode of action, acting as agonists (or partial agonists) of the serotonin (5-HT) receptor 5-HT_2A_, through which they exert their effects in the central nervous system (CNS) [1,6,11].

Ayahuasca, a Quechua word that means “vine of the souls” [4,12], and also known as *yagé*, *hoasca*, *caapi*, *daime*, and *natem* [13], is a hallucinogenic herbal preparation with a long traditional use both for therapeutic and divination purposes by indigenous tribes of the Amazon Basin, who consider it a sacred beverage [14]. The pharmacological activity is mostly attributed to the allegedly synergistic interaction between the psychoactive alkaloids of *Psychotria viridis* and of the *Banisteriopsis caapi* vine [12], the most commonly used admixture plants. *P. viridis* leaves contain high amounts of DMT, a potent short-acting psychedelic alkaloid that is inactivated *per os* due to first-pass metabolism by monoamine oxidase A (MAO-A) enzymes. β-Carboline alkaloids, such as harmine and harmaline, occur in the stem and bark of *B. caapi* and are potent and reversible inhibitors of MAO-A (MAO-AI), also having psychotropic properties [6,15]. Concomitant intake through ayahuasca allows the delivery of high levels of DMT to the CNS, enabling a potent psychotropic action [16]. Stephen Szara extracted DMT from another herbal constituent of ayahuasca preparations, i.e., *Mimosa hostilis*, and self-administered 1 mg/kg extract through intramuscular injection, after noting that *per os* administration of DMT did not lead to any noticeable effects [2,17]. As first reported by this Hungarian chemist and psychiatrist [17], DMT psychotropic effects include euphoria, visual hallucinations, spatial distortions, and speech disturbance, very similar to what had been previously described for LSD [18].

The two syncretic Brazilian churches *União do Vegetal* (UDV) and *Santo Daime* have an historic use of ayahuasca as a sacrament in religious ceremonies [12]. Such religious groups use ayahuasca both as a healing tool and as a way to “get in touch with the divine realm” [19]. The growing number of religious institutions as well as centres of alternative therapies that are allowed to use ayahuasca, led to a worldwide spread consumption by users seeking a spiritual experience or a direct psychedelic effect [20,21,22]. As such, its recreational use is sharply rising, with a global scale online survey showing an increased popularity on DMT consumption [23]. Such growing interest urges the understanding of the overall pharmacological action and safety of ayahuasca and its single bioactives. Furthermore, ayahuasca has also been brought into the spotlight by researchers due to the potential therapeutic benefits deriving from the modulation of the serotonergic system.

Herein, we intend to comprehensively review the chemistry, toxicokinetic and toxicodynamic aspects of ayahuasca and its active alkaloids DMT and the β-carboline/harmala alkaloids harmine, harmaline, and tetrahydroharmine (THH). As such, the main psychological and physiological modifications mediated by ayahuasca will be addressed, covering also the available data on toxic effects as well as on the pharmacology that might underlie a possible therapeutic use. A brief mention on the forensic relevance and regulatory status of ayahuasca and its psychoactive constituents will be also included.

## 2. Methodology

Articles dealing with the pharmacology, toxicity, therapeutic potential, metabolism, and forensic context of ayahuasca and its active alkaloids (DMT and β-carbolines) were identified through an English extensive literature search carried out in PubMed (U.S. National Library of Medicine) and Scopus, without a limiting period of time. Books or sections of books have been also included.

## 3. Plant Sources and Bioactives

The most common recipe of ayahuasca involves the combination of the leaves of *P. viridis* (commonly known as *chacruna*) with the malpighiaceous Amazonian jungle liana *B. caapi* (also commonly called ayahuasca) stem and/or bark [12,14], the latter being commonly used in the preparation of a wide range of herbal preparations. However, this psychotropic aqueous concoction could be obtained from 90 different admixture plants used by indigenous groups inhabiting the Amazon rainforest [24]. For example, in Colombian Putumayo and Ecuador, the leaves of *Diplopterys cabrerana* (also known as *chaliponga*) are used instead of *P. viridis* [12]; in Peru, several plants are frequently added to the aqueous concoction of *B. caapi* and *Psychotria* spp., most commonly *Nicotiana tabacum* (tobacco), *Brugmansia* spp., and *Brunfelsia* spp. [12,25].

**Figure 1:**
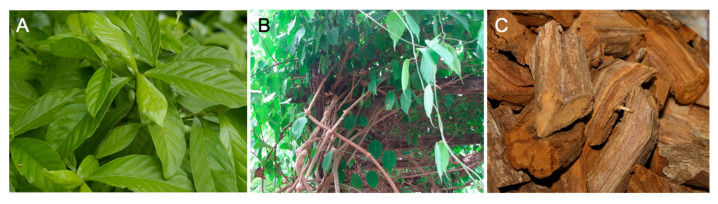
*Psychotria viridis* (**A**) and *Banisteriopsis caapi* (**B**,**C**), the most common admixture plant species used in ayahuasca preparations.

The main active alkaloids underlying the psychoactive effects of ayahuasca were first characterised by Rivier and Lindgren [26]. DMT occurs in the leaves of *P. viridis* [26,27], while *B. caapi* bark accumulates three main active constituents: harmine, harmaline, and THH [16,27,28,29] (Figure 1).

DMT, the main psychoactive constituent of ayahuasca, has long been classified as a structurally simple “spirit molecule” [2], occurring in over 50 plants from South American flora [24], including, but not limited to, *P. viridis*, *Desmanthus illinoensis*, *M. hostilis*, and *Phalaris aquatica* [30,31]. It is also an ubiquitous endogenous constituent of mammalian species, including humans, being detected in blood, urine, and cerebrospinal fluid [32,33,34].

Main active alkaloids occurring in *B. caapi* can be called “β-carbolines”, due to the shared tricyclic β-carboline structure, or “harmala alkaloids”, since harmine was first isolated from *Peganum harmala* [35]. Besides plants, these alkaloids are also present in animals, including humans, and many fungi [12,36].

The content of DMT in *P. viridis* ranges from 0.1% to 0.66% dry weight, while the β-carboline alkaloids content in *B. caapi* ranges from 0.05% to 1.95% dry weight [16,26]. Besides phytogeographical factors, the content of the alkaloids present in ayahuasca preparations (Table 1) frequently varies according to the selected plants [37], but also due to different extraction procedures [12,38]. Content determination in aqueous preparations of ayahuasca from Rio Purús revealed the presence of 65 mg alkaloid per 200 mL ayahuasca, consisting of average values of 30 mg of harmine, 10 mg of THH, and 25 mg of DMT [26]. A higher content was determined in samples from the UDV by Callaway [13]; accordingly, 100 mL of concoction contained 170 mg of harmine, 20 mg of harmaline, 107 mg of THH, and 24 mg of DMT. Values exceeding those were reported by McKenna et al. [16] after analysing five samples from Pucallpa, where a 100 mL dose of ayahuasca contained 467 mg of harmine, 160 mg of THH, 41 mg of harmaline, and 60 mg of DMT.

## 4. Prevalence and Patterns of Use

Ayahuasca is mainly used in religious contexts in ceremonials organised by syncretic churches, with the large ones being the *Santo Daime* and the UDV, with about 10,000 attendees each [12]. A smaller church, the Barquinia, had also employed ayahuasca into their religious practices. Its consumption is done weakly or bimonthly in a ritual manner similar to the Christian Eucharist [12,13,39]. There have been few reports on the consumption of ayahuasca by children and pregnant women belonging to *Santo Daime* and UDV churches [40]. Children with <12 years can only participate in 5 or less rituals per year; they are allowed to participate once a month if they are between 12 and 14 years; twice a month with 14 to 18 years; and they may become full members of the church with >18 years [40].

In the last two decades, ayahuasca use has spread throughout the urban centres of South and North America, Europe, Asia, and Africa [30,41,42,43], leading to increased popular interest in this brew as well as in DMT. In fact, a lot of foreigners travel to the Amazon (called “ayahuasca tourism”) to participate in ceremonial rituals organised by indigenous people [30]. There is also an increased search of online “headshops” that legally trade-in ayahuasca ingredients [44], such as plants rich in DMT and harmala alkaloids, making them widely available to users.

An online survey aiming to explore the pattern of recreational DMT use and identify the users’ demographics, was conducted by Cakic et al. [45] between July and August 2009 with Australian residents. Lifetime DMT users accounted for a total of 121 individuals; 86.8% were male and the median users age was 28 years. Those reported having no religious affiliation were 73.6%, 60.3% had completed a university degree, and 10.7% were unemployed. The median age for the onset of DMT use was 24 years; the median total number of occasions a participant used DMT was 10 times, and median duration of DMT use was 2 years. Smoking was the most common route of DMT administration (98.3%), although some also reported using DMT *per os* in the form of ayahuasca (30.6%), while insufflation (5%) and injection (2.5%) were less common routes of administration. The respondents first heard about DMT through friends (47.9%), the Internet (24.8%), and print media (22.3%). 60.3% obtained or purchased DMT from friends, and 26.4% extracted DMT themselves from plant materials, which was reported by 30.6% of the survey participants as being somewhat easier to obtain/purchase. The median reported price for 1 g DMT was US$150, as it was somewhat difficult (36.4%) to obtain. Almost all participants reported using DMT in their own home (81.8%) and in a small group of up to four friends (76.9%). Among the DMT smokers, 68.1% reported concomitant use with other drugs, including cannabis, LSD, alcohol, psilocybin, and 3,4-methylenedioxymethamphetamine (MDMA); and among ayahuasca users, 27% also reported the co-use of other drugs (including cannabis and psilocybin).

Winstock et al. [23] studied the prevalence of DMT use through anonymous online surveys conducted between November and December 2012, within a large contemporary global population of drug users. A total of 22,289 responses were received, which included 7784 (34.9%) from Australia, 7360 (33.0%) from the United Kingdom (UK), 3756 (16.9%) from US, and 2164 (9.7%) from Europe. An interesting result was that at least 472 (2.1%) of the total respondents stated that DMT was the last new drug they had tried. 1980 (8.9%) reported the use of DMT at least once in their lifetime, 1123 (5.0%) in the past year, and 363 (1.6%) in the last month. In this study, the demographic characteristics of DMT users were also determined: 61.7% were male and 29.7% female; mean age was 32 years; 87.4% were Caucasian, 0.5% Negroid, 2.3% were Asian, 3.1% had mixed ethnicity, and 2.5% had other ethnicity; 77.2% were heterosexual, 7.8% homosexual, and 9.0% bisexual; mean wellbeing score was 56.2; 67.9% said to be working, 35.8% studying, and 24.3% unemployed. Considering the routes of administration, 2.1% of DMT users reported snorting, 3.0% swallowing, 92.2% smoking, 2.8% using other routes of administration, and no respondent said they injected DMT. Contrary to this pattern, ketamine users tend to prefer snorting (89.0%), while LSD and magic mushrooms users orally ingest the drugs (87.8% and 89.6%, respectively).

During 2014–2016, Kaasik and Kreegipuu [46] conducted a study based on questionnaires, aiming to describe the patterns of ayahuasca use in Estonia. Thirty ayahuasca users (50% male and 50% female) were included, with an average age of 38.7 years (age range 25–62). Demographic data showed that only 6 (20%) ayahuasca users do not have a university degree, with 3 (10%) having a Ph.D.; 43% were employed, with 47% being entrepreneurs; and 70% lived in an urban environment. Of the 30 ayahuasca users, only 4 said that they will “probably not” use ayahuasca in the future. The first time they used ayahuasca in Estonia was in 2002–2013, 2008 and 2010 being the years with the higher number of first users. The use of ayahuasca occurred mostly during group private ceremonies, or individually for self-medication or exploration, with no reports of recreational or public use. Participants participated in a median of 10 ceremonies (ranging from 1–250), and the average age of the onset of ayahuasca use was 33.4 years (range 21–58). Information about the ceremonies’ occurrence was shared by e-mail and personal communication, with the inexistence of public announcements. Both before and after the ceremonies, participants reported going through a period of restrictions, namely restraint of certain foods and medications, and sexual abstinence.

## 5. Legal Status

Ayahuasca is officially recognised, protected, and legally permitted by the Brazilian regulatory agency *Conselho Nacional de Políticas sobre Drogas* in the context of religious ceremonies performed by established churches [12], its use by the *Santo Daime* also being legally allowed in Canada [43]. In addition, it benefited in 2006 from the decision made by the US Supreme Court that established the protection of its religious use in the US under the 1993 Religious Freedom Restoration Act [45,47]. Despite such permissions, ayahuasca is illegal in the US, Canada, The Netherlands, and France [48]. In Portugal, despite its possession in small amounts for self-use being decriminalised by the law 30/2000, November 29, the sale, transport, and cultivation of ayahuasca plants is illegal [48].

DMT is illegal in most countries, being classified as a Schedule I drug by the United Nations (UN) 1971 Convention on Psychotropic Substances [49]. It is categorised as a Class A substance in the UK, as a Schedule I hallucinogenic substance by the Drug Enforcement Agency in the US [6,23,50,51], as a Schedule III drug in Canada, a controlled substance in France and Portugal (the drug is included in the Table II-A of the Decree Law n^o^ 15/93), and as a Schedule I drug under the German Narcotics Act; it is also banned in Japan [48,52].

The harmala alkaloids are also regulated in a few countries, although not being subject to international control. In 2005, France added harmine, harmaline, THH, harmol, and harmalol to the list of controlled substances (*Journal Officiel de la République Française* n^o^ 102 du 3 mai 2005; NOR: SANP0521544A), being, to the best of our knowledge, the only country in Europe where these substances are illegal. In Canada, harmalol and harmaline are also classified as Schedule III drugs under the 1996 Controlled Drugs and Substances Act.

On the other hand, the herbal products used for ayahuasca preparation, and known to contain Schedule I international controlled substances like DMT, are not subject to international control and have a lack of legal control in most countries [22,49]. An exception to this includes France, where non-licensed possession of *P. viridis*, *B. caapi*, *P. harmala*, and other plants containing DMT/harmala alkaloids have been banned since 2005 (*Journal Officiel de la République Française* n^o^ 102 du 3 mai 2005; NOR: SANP0521544A); and countries where all psychoactive compounds-containing plants are illegal, like Russia [53].

## 6. Structure and Physicochemical Properties

**Figure 2:**
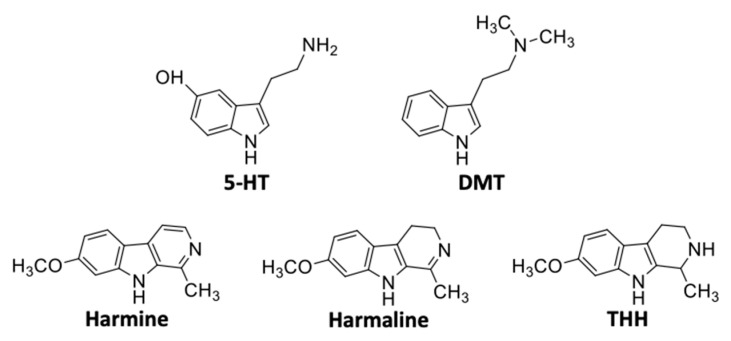
Chemical structure of serotonin (5-HT) and the main active alkaloids present in ayahuasca preparation. DMT: *N*,*N*-Dimethyltryptamine; THH: Tetrahydroharmine.

Deriving from decarboxylation of the biosynthetic precursor tryptophan via the aromatic L-amino acid decarboxylase, tryptamine is subsequently *N*,*N*-demethylated by indolethylamine-*N*-methyltransferase, *S*-adenosylmethionine serving as the methyl donor, ultimately leading to *N*-methyltryptamine (NMT) and DMT [54,55]. DMT is structurally similar to melatonin and the neurotransmitter 5-HT, the latter playing a pivotal role in the modulation of human mood and behaviour [56]. While sharing the tryptamine core, DMT bears a particular feature, i.e., the *N*,*N*-dimethyl moiety (Figure 2) [4]. The structural backbone is also similar to that of the triptan class of vasoconstrictors, clinically used to treat migraines and cluster headaches [57]. Such structural similarity suggests that slight modifications to the DMT molecule can enable the development of synthetic analogues lacking hallucinogenic properties, but with potential therapeutic utility [56]. On the other hand, minor modifications and/or substitutions frequently maintain a psychedelic ability as demonstrated by several serotonergic psychedelics, such as the 4-substituted psilocybin and psilocin, 5-methoxy-*N*,*N*-dimethyltryptamine (5-MeO-DMT) and 5-methoxy-*N*,*N*-diisopropyltryptamine (5-MeO-DIPT) [4].

DMT is a lipophilic molecule (logP 2.573) with a rather small structural backbone [molecular weight (MW) 188.27 g/mol]. In its freebase form (commonly used for inhalation), DMT can be seen as clear or white crystals. It has a melting point (Mp) of 44.6 °C to 46.8 °C, and a pK_a_ value of 8.68, being only soluble in diluted acetic acid and diluted mineral acid. DMT hydrochloride is a white crystalline powder soluble in water; it has a Mp of 165 °C to 168 °C, a pK_a_ of 8.7, and a LogP of 1.9 [58]. DMT fumarate (MW of 304.34 g/mol) is a water-soluble salt form of DMT, commonly used for drug administration by injection, and it is more stable for long-term storage than the freebase. In solution, DMT has a fast degradation rate and should be stored at −20 °C, protected from air and light. Additionally, under certain conditions, i.e., elevated heat, it can have explosive potential [59].

Harmala alkaloids (Figure 2) are biosynthesised from tryptophan and/or tryptamine, through a condensation process involving indolamines along with aldehydes or α-keto acids [12]. Harmine appears in crystal form, having a MW of 212.25 g/mol, and a Mp of 261 °C with decomposition or sublimation. It is soluble in organic solvents such as ethanol, chloroform, and ether, with a LogP of 3.56 and a pK_a_ of 7.6 [58]. Harmaline has a MW of 214.3 g/mol and a Mp of 249–250 °C. It is slightly soluble in ethanol and ether, with a LogP of 2.1 [58,60]. THH has a MW similar to that of harmaline and harmine (216.28 g/mol) and a LogP value of 1.9, being soluble in ethanol, chloroform, and ethyl acetate [61]. A summary of the physicochemical properties of DMT, harmine, harmaline, and THH is presented in Table 2.

## 7. Pharmacokinetics

### 7.1. Routes of Administration and Absorption

Independently of the doses used (which are as high as 13 mg/kg), pure DMT administered *per os* is unable to lead to psychotropic activity due to rapid and extensive first pass metabolism, culminating in very low bioavailability [62,63]. Administration through other routes evidences that both serum concentrations and effects produced by DMT can greatly vary [38].

Under recreational consumption, DMT freebase is typically smoked or nasally insufflated, although intravenous (i.v.) injection is also widely used for DMT fumarate salts [1,38]. Typical doses for smoked DMT are 40−50 mg (approximately 0.6–0.7 mg/kg), although doses as high as 100 mg may be used (approximately 1.4 mg/kg), while for injected DMT, usual doses range from 0.1 to 0.4 mg/kg [31,64]. Doses as low as 0.2 mg/kg, both through inhalation or i.v. administration, appear to enable the hallucinogenic effects [65,66]. Smoked or inhaled DMT freebase has a rapid and overwhelming action onset, with full hallucinogenic manifestations beginning immediately after administration, typically at 10–15 sec, peaking at 5 min, and having a very short duration that lasts for less than 1 h [1,37]. Unlike other psychedelics leading to long-lasting effects (e.g., 4–6 h for psilocybin; 12 h for LSD and mescaline; and 24 h for ibogaine), smoked DMT causes a short-term but potent effect, being coined as the “businessman’s lunch trip” in the 1960s [56]. Similar effects have been reported after i.v. administration of water-soluble fumarate salt of DMT [64,67].

Available data on the pharmacokinetics of the harmala alkaloids as single entities is scarce. In a study by Plutarco Naranjo Vargas in the 1960s [68], following oral administration of 20–50 mg of harmine to humans (approximately 0.3–0.7 mg/kg), the onset of psychotropic action appears to occur at 20–30 min, with the peak happening at 30 min to 1 h, and lasting up to 6–8 h. When 10–20 mg (approximately 0.1–0.3 mg/kg) is administered through i.m. injection, the reported onset of effects was at 5–10 min, with a maximum effect reached at 30 min, and lasting up to 3–5 h [68]. In Wistar rats, 24 h after oral administration of total alkaloid extracts from seeds of *P. harmala* at doses of 15, 45, and 150 mg/kg, both harmaline and harmine were shown to be rapidly absorbed into the bloodstream [69]. Maximum plasma concentrations (Cmax) displayed a dose-dependent increase, and were obtained at the times (Tmax) 0.73–4 h for harmaline and 0.69–2.7 h for harmine. After oral administration of 40 mg/kg of harmaline and harmine to Sprague-Dawley rats, absorption to blood circulation occurred with an approximately Tmax of 1.76 h for harmaline and of 0.56 h for harmine [70]. As shown in this study, oral bioavailability of harmaline (17.11%) is higher than that of harmine (1.09%).

Both the onset and duration of the hallucinogenic effects are altered when DMT and β-carbolines are co-administered in ayahuasca. Following oral intake, the peak of action occurs at 1.5 to 2 h, and the hallucinogenic state can last for approximately 4 to 6 h [45,71]. While this fits nicely with DMT plasma concentrations [35], the same does not occur with the β-carbolines, since the peak plasma concentrations of harmaline and THH appear after the effects have occurred. This further supports the reports on DMT as the main psychoactive constituent of ayahuasca [35]. The time of the peak plasma concentrations of DMT has been reported to range from 1.5 to 1.8 h, following ayahuasca consumption [13,72,73]. Callaway [13] further reported Tmax values for harmine (1.7 h), harmaline (2.4 h), and THH (2.9 h). Riba et al. [73] recorded plasma concentrations after the oral intake of low (0.6 mg/kg of DMT, 1.0 mg/kg of harmine, 0.07 mg/kg of harmaline, and 0.82 mg/kg of THH) and high (0.85 mg/kg of DMT, 1.4 mg/kg of harmine, 0.09 mg/kg of harmaline, and 1.16 mg/kg of THH) doses of ayahuasca. Although harmine was undetected, its metabolite harmol had a peak concentration in plasma at 1.5 h at the low dose, and at 2 h at the high dose. Similar Tmax values were obtained for harmaline, while those of THH were higher, being at 2.5 and 3 h after low and high doses, respectively. Harmalol, the *O*-demethylated metabolite of harmaline, was also quantified with Tmax of 2.5 and 2.75 h after low and high doses, respectively. In these studies, a trend for increase in Tmax values from DMT to harmaline and THH was seen.

### 7.2. Distribution

DMT is rapidly distributed following intraperitoneal (i.p.) administration of 10 mg/kg in adult male Sprague-Dawley rats, with maximum concentrations in the liver and kidney occurring 5 min after administration, and in the brain after 10 min, followed by a complete clearance from all tissues within a period of 30–70 min [74]. Cohen and Vogel [75] also reported in rats, following administration of 5 mg/kg of DMT, the rapid distribution from the i.p. cavity through plasma (0.4 mg/kg), liver (6.8 mg/kg), and brain (0.9 mg/kg), where it could be detected 5 min after administration, maximum concentrations occurring at 5 and 10 min in the liver (6.8 mg/kg) and at 15 min in the brain (1.8 mg/kg). At 30 min, almost no DMT could be detected in these tissues, especially in the brain, probably as a consequence of its fast metabolism and excretion. Due to its lipophilic profile and rather small structural backbone, DMT easily crosses the blood-brain barrier (BBB) [56]. DMT might be also transported into the rat brain through an active uptake mechanism, highly accumulating in the cortex and amygdala, pivotal structures underlying the behavioural effects of the drug [76,77]. Variations in plasma levels are consistent with the time course of the hallucinogenic effects [78], concentrations of DMT in the brain generally coinciding with the induced effects [79]. Strassman and Qualls [65] reported that DMT was not detected in blood samples after 1 h following i.v. administration, similar findings being reported for intramuscular (i.m.) injection [78].

Wang et al. [69] evaluated the tissue distribution of harmine and harmaline after oral administration to Wistar rats of total alkaloid extracts from seeds of *P. harmala* for four weeks, at daily doses of 15, 45, and 150 mg/kg. Concentrations in the analysed tissues showed a dose-dependent increase; both harmaline and harmine were widely distributed, with higher concentrations found in the liver (up to approximately 0.55 mg/kg for harmaline and 0.05 mg/kg for harmine), kidney (up to approximately 0.15 mg/kg for harmaline and 0.045 mg/kg for harmine), spleen (up to approximately 0.1 mg/kg for harmaline and 0.035 mg/kg for harmine), and lung (up to approximately 0.15 mg/kg for harmaline and 0.035 mg/kg for harmine), without accumulation. Only harmaline could be found in the brain, at a maximum concentration of approximately 0.05 mg/kg, suggesting an ability to cross the BBB.

### 7.3. Metabolism

**Figure 3:**
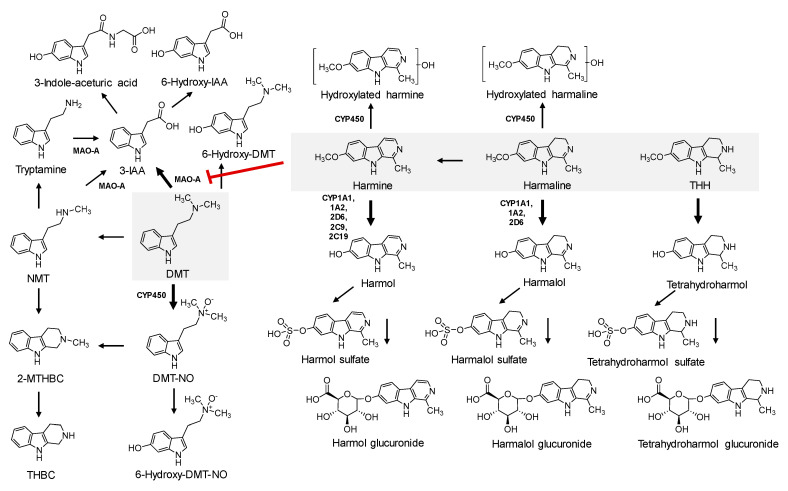
Metabolic pathways of *N*,*N*-dimethyltryptamine (DMT) and of β-carboline alkaloids harmine, harmaline, and tetrahydroharmine. The influence of β-carbolines on the metabolism of DMT is also represented in red. 2-MTHBC: 2-Methyl-1,2,3,4-tetrahydro-β-carboline; 3-IAA: 3-Indole-acetic acid; DMT-NO: DMT-*N*-oxide; NMT: *N*-Methyltryptamine; THBC: 1,2,3,4-Tetrahydro-β-carboline.

Metabolic pathways of both DMT and harmala alkaloids are represented in Figure 3. After oral administration, DMT undergoes rapid and extensive oxidative deamination, mediated by visceral MAO-A enzymes, to the inactive metabolite 3-indole-acetic acid (3-IAA) contributing to the reduced bioavailability [73,80] and explains the absence of both hallucinogenic effects and DMT in the urine of consumers [17,78,81]. MAO enzymes are present in the mitochondrial membrane, mainly distributed in the blood, liver, kidney, spleen, stomach, brain, and intestines [82]. While both isoforms (MAO-A and MAO-B) are responsible for the oxidative deamination of endogenous and exogenous amine substrates and the modification of amino acids within proteins [83,84], serotonin and related tryptamines such as DMT are specific substrates of MAO-A [38]. In contrast with the visceral enzyme, plasma circulating MAO-A deaminates only primary amines [85], and therefore parenterally administered DMT can reach CNS and induce psychotropic effects. Notwithstanding, DMT rapidly reaches mammalian tissues after parenteral administration, namely the brain, where it is also promptly metabolised by MAO-A [86], explaining the rapid onset and short duration of hallucinogenic effects after an administration by smoking or injection [17].

3-IAA and 3-indole-aceturic acid were reported as the main urinary metabolites of DMT in rats [81]. Alternative metabolic pathways have been described both in vitro and in vivo, which include *N*-oxidation, *N*-demethylation, and cyclization [27,74,87,88]. The second main metabolic pathway renders DMT-*N*-oxide (DMT-NO) through *N*-oxidation mediated by cytochrome P450 (CYP), studies reporting high concentrations of the metabolite in human blood and urine after DMT ingestion with MAO-AI [27,80,89]. DMT-NO retains a closer structural similarity with the parent compound [90], although not being a substrate for MAO [88]. A negative correlation between IAA/DMT-NO ratio and the presence of psychoactive effects was inclusively found by Riba et al. [91], reaffirming the quantitative significance of this metabolite. In addition, DMT can be *N*-demethylated to form NMT, which is a minor metabolite and acts as a MAO-A substrate, therefore likely to be further metabolised into IAA [80]. During the metabolisation of DMT, trace amounts of tryptamine and tetrahydro-β-carbolines are also formed, namely 2-methyl-1,2,3,4-tetrahydro-β-carboline (2-MTHBC) and 1,2,3,4-tetrahydro-β-carboline (THBC) [87]. 2-MTHBC derives from the cyclization either of DMT-NO or NMT. There are also reports on 6-hydroxy-DMT, 6-hydroxy-DMT-*N*-oxide and 6-hydroxy-indole-acetic acid as additional trace metabolites [92].

Sitaram et al. [74,90] demonstrated that, after pre-treatment with the MAOI iproniazid, concentrations of unmetabolised DMT and the main derivatives (DMT-NO and NMT) were found to be effectively increased in rat tissues (brain, liver, kidney, and blood), persisting 45 min after drug intake, and also found to be increased in urine. This further supports a shift towards these secondary metabolic pathways, after pre-treatment with MAO-AI. The CYP-dependent metabolism is less efficient and active than the MAO-mediated oxidative deamination. Consequently, DMT breakdown is slower and less extensive [91], culminating in higher amounts of the parent drug accessing the bloodstream and reaching the CNS [56]. Increased and long-term effects of DMT [4] are also due to the decrease of MAO-mediated metabolism in the brain.

The β-carboline alkaloids harmine and harmaline, and to a lesser extent THH, reversibly inhibit MAO [38]. After ayahuasca consumption, the inhibition of the MAO-A causes reduced deamination of DMT, and a shift to the alternative metabolic pathways as a compensatory metabolic mechanism [80]. As such, after ayahuasca administration, higher amounts of DMT-NO and NMT are formed [27].

CYP450 complex is also responsible for the metabolisation of β-carboline alkaloids, which is mainly active in the liver [93]. Harmaline is *O*-demethylated into harmalol by CYP1A1, CYP1A2, and CYP2D6, harmine being metabolised into harmol by these isoforms but also by CYP2C9 and CYP2C19 [22]. CYP1A2 and CYP2D6 are suggested to be the major CYP450 isoenzymes responsible for the catalysation [94]. Harmaline and harmine were also reported to be hydroxylated through CYP450 [15,95]; additionally, harmaline can suffer dehydrogenation into harmine [15]. Harmalol and harmol are further metabolised through phase II metabolism, being excreted as glucuronic and/or sulphate conjugates, with a reported predominance of the glucuronides in humans [15,95]. THH can also be *O*-demethylated to tetrahydroharmol (7-hydroxy-tetrahydroharmine) [80].

### 7.4. Excretion

After i.p. injection of DMT to rats, Sitaram et al. [74] estimated the in vivo half-life (t_1/2_) of elimination from the brain (5.7 min), liver (9.6 min), kidney (17.2 min), and blood (15.8 min). The same research group further reported that 90% of the total amount of DMT and its congener 5-MeO-DMT, a tryptamine derivative with lower affinity for MAO-A, was excreted in urine during the first 3.5 h, following i.p. injection [90], coinciding with rapid metabolism and excretion. After oral self-administration of 0.38 mg/kg DMT by 6 individuals with previous experience taking this drug, no parent drug could be recovered from 24-h urine; 3-IAA accounted for 97% of the dose, with the remaining 3% corresponding to DMT-NO, suggesting a rapid breakdown [17]. However, when the same dose was smoked, 10% of the recovered analytes in urine corresponded to unmetabolised DMT, 28% to DMT-NO, and 3-IAA accounted for 63%. Sitaram et al. [90] suggested extensive DMT metabolisation prior to excretion, as following an i.p. administration of 10 mg/kg DMT to rats, less than 1.1% of the administered dose could be recovered from urine as parent compound, whereas 6.5% and 0.02% were recovered as DMT-NO and NMT, respectively. After pre-treatment with a MAOI (100 mg/kg of iproniazid phosphate), an increase of DMT (accounting for 2.1% of the administered dose) and its structural characteristic metabolites (20.6% of DMT-NO and 0.6% of NMT) was seen in urine [90].

DMT elimination can also be prolonged with the co-consumption of MAOI, as those produced by *B. caapi*. In a study enrolling 15 volunteers who ingested a single dose of ayahuasca (2 mL/kg, corresponding to 0.48 mg/kg of DMT, 3.4 mg/kg of harmine, 0.4 mg/kg of harmaline, and 2.14 mg/kg of THH) [13], a DMT t_1/2_ of approximately 4 h, t_1/2_ values of 2 and 8.9 h were recorded for harmine and THH, respectively. Riba et al. [73] studied 18 volunteers after receiving low (0.6 mg/kg of DMT, 1.0 mg/kg of harmine, 0.07 mg/kg of harmaline, and 0.82 mg/kg of THH) and high (0.85 mg/kg of DMT, 1.4 mg/kg of harmine, 0.09 mg/kg of harmaline, and 1.16 mg/kg of THH) oral doses of ayahuasca, and reported lower DMT t_1/2_ of approximately 1.07 h, explained by a lower degree of MAO inhibition related with a lower content of harmala alkaloids in the samples under study. In fact, the authors [73] further reported that lower t_1/2_ values were also obtained for the other alkaloids— 2.01 and 1.95 h for harmaline, 4.78 and 4.68 h for THH, 1.64 and 1.49 h for harmol, and 30.33 and 48.64 h for harmalol—after the low and high doses of ayahuasca, respectively. Harmine was undetectable in the plasma of some users, suggesting that the alkaloid is readily metabolised before reaching systemic circulation [73]. Although this is the main MAOI present in ayahuasca, participants still reported common psychoactive effects, suggesting that partial MAO inhibition, being peripheral and short-lived, is enough for DMT to reach systemic circulation and subsequently exert its effects in the CNS [35,73].

Riba et al. [80] determined the 24 h-urinary disposition of DMT and the harmala alkaloids, after ingestion of ayahuasca obtained from a Brazilian batch of 8.33 mg/g DMT, 14.13 mg/g harmine, 0.96 mg/g harmaline and 11.36 mg/g THH, by 10 young male healthy volunteers in doses equivalent to 1.0 mg/kg of DMT. Less than 1% of the unmetabolised drug was recovered; 3-IAA was the major metabolite found in urine with a recovery of ca. 50%; DMT-NO was the second major metabolite with recoveries around 10%, while 2-MTHBC and NMT accounted for only 0.2% of the administered dose. The high amounts of 3-IAA having been determined in the first 4 h following ayahuasca intake suggest that MAO inhibition was either incomplete or short-lived, nevertheless sufficient to allow DMT central effects. Considering their low urine recoveries, harmala alkaloids also appear to be extensively metabolised [80]. Harmaline had a higher recovery (8.5% of the administered dose), although THH was the main β-carboline found in urine (6.6% recovered). Harmalol was the *O*-demethylated metabolite with the higher recovery, ranging from 18% to 57%. Tetrahydroharmol was the most abundant metabolite in urine before enzymatic treatment, while after β-glucuronidase/sulfatase hydrolysis, the amounts of harmol in urine were higher than harmalol and tetrahydroharmol. Mcilhenny et al. [27] determined the concentrations of these compounds excreted in the urine of volunteers, following ayahuasca administration (0.75 mg/kg of DMT), without accounting for 3-IAA. DMT-NO was the major metabolite found in urine, with a peak concentration at 4–8 h (11 μg/mL). Significant peak concentrations of harmalol and harmol were also reported (4 μg/mL and 3 μg/mL respectively, at 4–8 h), which were significantly increased after enzymatic treatment with β-glucuronidase/sulfatase (11 μg/mL of harmalol and 115.49 μg/mL of harmol, at 4–8 h), although peak concentrations were obtained at 0–4 h after treatment (14.16 μg/mL of harmalol and 126.18 μg/mL of harmol). THH had a peak concentration at 4–8 h (6.27 μg/mL), being the major excreted compound (higher than 5 μg/mL) when considering the 8–24-h interval. Much lesser amounts in urine were observed for the other compounds, as demonstrated by the peak concentrations obtained for harmaline (0.51 μg/mL at 0–4 h), harmine (0.16 μg/mL at 0–4 h), 2-MTHBC (0.13 μg/mL at 4–8 h), and unmetabolised DMT (0.6 μg/mL at 4–8 h).

In Table 3, a summary of the pharmacokinetic properties of the ayahuasca alkaloids DMT, harmine, harmaline, and THH is presented.

## 8. Pharmacodynamics

It has long been believed that the effects of serotonin were mimicked by psychedelic drugs [2]. In fact, classic hallucinogens like DMT act as agonists of serotonin receptors, leading to increased brain levels of serotonin [96,97,98]. Preliminary findings [99] suggested that hallucinogenic drugs exert their effects by acting on a specific group of serotonin receptors, the 5-HT_2_ receptors. Subsequent studies further corroborated the involvement of the serotonin 5-HT_2A_ receptor in the visual hallucinations caused by classic psychedelics [6,100], which are nowadays considered a key site for their action [1].

DMT has affinity for a variety of neuroreceptors, including the types 1A, 1B, 1D, 2A, 2B, 2C, 6 and 7 of 5-HT receptor, with reports of at least a partial agonism of the 5-HT_1A_ (K_i_ = 183 nM), 5-HT_2A_ (K_i_ = 127 nM), and 5-HT_2C_ (K_i_ = 360 nM) [56,101,102]. In a study with rat fibroblasts expressing the 5-HT_2A_ or the 5-HT_2C_ receptors, DMT was characterised as a full agonist of the former, but only a partial agonist of 5-HT_2C_, profound desensitisation over time only being observed with the 5-HT_2C_ receptor [102]. Contrary to other psychedelics, for which repeated administration leads to a very rapid development of tolerance [6], DMT does not appear to induce human tachyphylaxis [103]. In this sense, desensitisation of 5-HT_2C_ in response to repeated DMT administration in humans suggests that its mechanism of action is mainly mediated by the interaction with 5-HT_2A_ receptors. Additionally, the higher distribution of 5-HT_2A_ receptors in the cortex (the brain area where hallucinogens are thought to act) in comparison with 5-HT_2C_ reaffirms the higher relevance of 5-HT_2A_ receptors as mediators of psychedelic effects [104]. Of note, it has been suggested that the high affinity of DMT for 5-HT_2A_ receptors might derive from the small *N*-methylated moieties [105].

**Figure 4:**
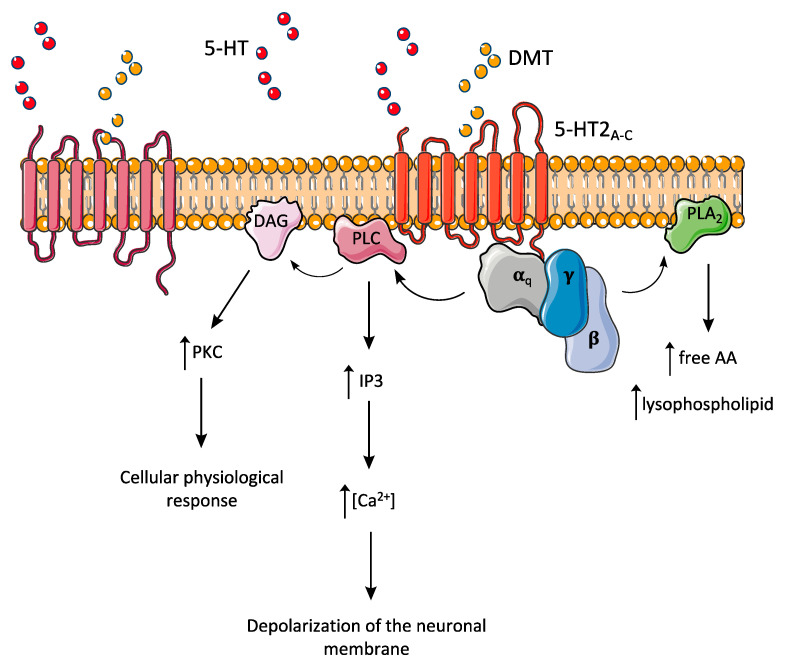
Main intracellular signalling pathways after activation of 5-HT2_A-C_ receptors by *N*,*N*-dimethyltryptamine (DMT). The main downstream signalling pathway of these receptors, for a long time assumed to be critical for the action of psychedelics [6], is coupled to Gq, resulting in the activation of phosphatidylinositol-specific phospholipase C (PLC) [6,106,107]. PLC hydrolyses phosphatidylinositol membrane lipids, resulting in the generation of inositol-1,4,5-triphosphate (IP3) and diacylglycerol (DAG) [108]. Whilst IP3 leads to calcium release from intracellular vesicles, resulting in depolarization of the neuronal membrane, DAG remains bound to the membrane and activates a second messenger, the protein kinase C (PKC), ultimately responsible for the mediation of the cellular physiological response. Another signalling pathway later identified, whose significance is still not well characterised [6], correspond to the stimulation of phospholipase A2 (PLA2), that consequently hydrolyses arachidonic acid (AA)-containing phospholipids, producing free AA and lysophospholipid [109]. 5-HT: Serotonin.

As most 5-HT receptors, 5-HT_2A_ is a member of the superfamily of G protein-coupled receptors [1], whose ligand-dependent stimulation leads to the activation of a series of intracellular signalling pathways (Figure 4) [6]. The receptor is found widespread in the mammalian brain throughout the cortex, striatum, hippocampus, and amygdala [106].

As reported in several studies, activation of 5-HT_2A_ by psychedelics can potentiate the expression of genes encoding transcription factors, such as *c*-fos, egr-1 and egr-2 [107,108], known to be associated with synaptic plasticity, memory, and attention [109,110,111]. Although the acute effects of DMT/ayahuasca or other psychedelics are related to the immediate electrophysiological changes after 5-HT_2A_ receptor activation, these longer-lasting implications in transcription modulation may trigger the differences observed in long-term users of ayahuasca, relative to brain structure and personality [35,112]. Furthermore, transcription modulation may underlie the known antidepressant effects of 5-HTergic psychedelics [113], including those suggested to derive from ayahuasca consumption [114,115].

The 5-HT_2C_ receptor is also coupled to G_q_, hydrolysis of phosphatidylinositol membrane lipids being increased after activation [102]. However, this agonist effect is not considered to play a main role in the production of DMT effects [102,116].

DMT is also an agonist of the pre-synaptic 5-HT_1A_ receptor [6]. This receptor is coupled to G_i_ proteins, being abundant in the somas and dendrites of serotonergic neurons in the raphe nuclei of the brainstem [35,117]. Contrary to 5-HT_2A/2C_, this receptor is involved in inhibitory neurotransmission, leading to reduced 5-HT release in other brain regions following its activation [56,118]. Desensitisation of these receptors can be achieved with chronic consumption of antidepressants, resulting in the restoration of the normal activity of 5-HT in neurons [119]. Due to this, the reported anxiolytic and antidepressant properties of ayahuasca and/or DMT [114] are thought to be also related with agonism towards 5-HT_1A_ receptors [56].

Little is known of the consequences of the interaction between DMT and the 5-HT_1D/6/7_ receptors. However, it would be important to evaluate the potential role of this interaction in the promotion of some behavioural and therapeutic effects of ayahuasca, since various aspects of learning, memory, neuroplasticity, and cognition, have been associated with 5-HT_6/7_ receptors [120,121,122,123].

Additionally, DMT is also known to act as a substrate of the vesicular monoamine transporter 2 (VMAT_2_) [124] and 5-HT transporter (SERT) [124,125]. As such, DMT effects may involve a complex and intricate interaction of multiple systems and cannot be solely explained by its action towards the 5-HT receptors. In fact, DMT also binds and activates non-serotonergic receptors [55]. DMT is one of the only known endogenous agonists of the sigma-1 receptor (σ1R) [126]. These receptors are expressed on the mitochondria-associated endoplasmic reticulum membrane of the brain, but also in lung, prostate, colon, ovary, breast, and liver cells [22]. After stimulation, sigma-1 receptor modulates calcium signalling through interaction with inositol-1,4,5-triphosphate (IP3) receptors at the endoplasmic reticulum membrane [127]. Additionally, it can migrate to the cell’s plasma membrane, where interaction with and inhibition of several ion channels, including voltage-gated sodium and potassium channels takes place [22,35,128]. The relation between the activation of this receptor and the psychedelic effects of DMT is, however, still debatable [6], as other substances that are devoid of hallucinogenic properties (e.g., cocaine) have also been known to exhibit a similar binding ability [129]. Since sigma-1 receptors have been targeted for the treatment of some neurological disorders [130,131], therapeutic properties of ayahuasca and/or DMT, including the treatment of depression, anxiety, schizophrenia, and promotion of neural plasticity, could be reasonably hypothesized to be, at least in part, associated with this receptor [56,132]. Their involvement in the antidepressant effects attributed to ayahuasca is supported by the mechanism of action of certain antidepressants (e.g., fluvoxamine) that also include the stimulation of sigma-1 receptors [35,133].

DMT also acts as an agonist at the trace amine-associated receptor type 1 (TAAR1), whose activation leads to an increase in cAMP production [134]. This activation was proposed to result in a suppression of psychosis and induction of a relaxed mental state [135]. The affinity of DMT to TAAR1 can be related to its anxiolytic properties [136], suggesting a potential therapeutic utility for DMT and ayahuasca.

**Figure 5:**
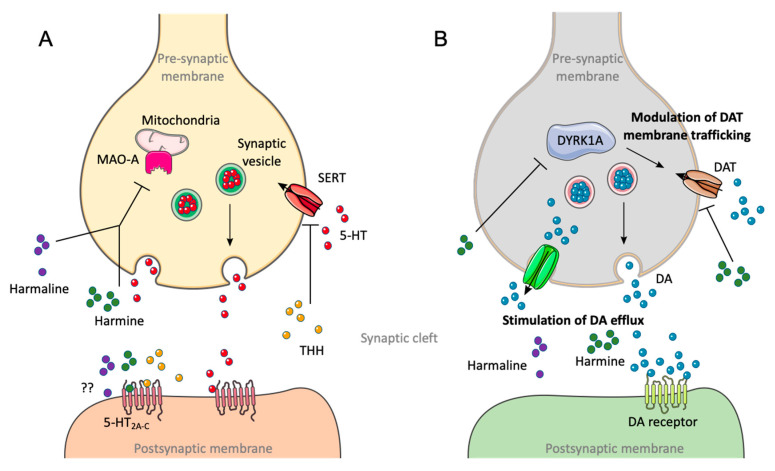
Pharmacodynamic mechanisms of harmine, harmaline, and tetrahydroharmine (THH) at the serotonin (5-HT) (**A**), and dopamine (DA) (**B**) pathways. DAT: DA transporter; DYRK1A: Tyrosine-phosphorylation-regulated kinase 1A; MAO-A: Monoamine oxidase A; SERT: 5-HT transporter.

Although DMT is considered to be the main alkaloid responsible for ayahuasca psychotropic properties, β-carbolines are also psychoactive, and, if in sufficient amount, may directly contribute to behavioural effects in consumers [137,138]. They can induce psychological and physiological effects via modulation of the levels of amine neurotransmitters in the CNS, either by inhibiting their metabolism or by direct interaction with specific receptors [22,139]. The β-carbolines harmine and harmaline act as selective and reversible MAO-AI, while THH acts as an inhibitor of the 5-HT reuptake (Figure 5) [140], with weaker or absent action upon MAO-A [13,29]. Hallucinogenic effects are also reported following the administration of β-carbolines without DMT, which could also be a result of their direct binding to 5-HT_2A_ or 5-HT_2C_ receptors [138,141].

Another main mechanism of action that has been proposed includes the stimulation of dopamine (DA) efflux (Figure 5) [142]. Accordingly, harmine and harmaline seem to cause increased DA release, affecting the pathways mediated by this neurotransmitter [22]. Less-studied mechanisms have also been proposed [22]. It was suggested that harmine inhibits the DA transporter (DAT) [143], resulting in high levels of DA in the synaptic cleft, thus modulating dopaminergic neurotransmission [144]. Additionally, harmine is a potent inhibitor of tyrosine-phosphorylation-regulated kinase 1A (DYRK1A), an enzyme responsible for modulation of DAT membrane trafficking (Figure 5), further corroborating the possible role of harmine in the rate of DA reuptake [142].

Ayahuasca also seems to induce changes in the concentration of the inhibitory neurotransmitter γ-aminobutyric acid (GABA), apparently deriving from psychoactive alkaloid constituents [145]. The opposite effects found between the hippocampus (excitation due to decrease GABA release) and amygdala (inhibition due to increased GABA release) suggest the modulation of several important pathways involved in memory, learning, and emotional behaviour.

## 9. Psychological and Physiological Effects

Depending on the dose, participant’s expectations, and disposition, and the setting where the consumption takes place, positive and adverse effects of DMT and ayahuasca can encompass a considerable degree of unpredictability [37]. DMT is thought to have dose-dependent effects in humans, visual hallucinations predominating at high doses, while stimulant effects are mostly noted at low doses [66]. After acute ayahuasca ingestion, psychoactive effects such as intense perceptual and cognitive changes, somatic effects, increased emotional liability, and positive mood are rapidly felt, being resolved within a maximum of 4 to 6 h [71,72,73]. Such consumption also results in moderate and transitory cardiovascular, autonomic, neuroendocrine, and immunological effects, being well tolerated when consumed by healthy individuals.

A cycle of experiences designated as the “transcendental circle” by Kjellgren et al. [146] are consistent among different individuals, following ayahuasca consumption. Changes in perceptions, visual field vibrating, and users feeling vulnerable are noted 30 min after ingestion, an experience known as the visionary state. Following this, terrifying feelings of confusion, paranoia, and fear can be experienced, which might be accompanied by nausea or vomiting. Then, participants usually mention contact with a spiritual world, characterised by feelings of oneness with the universe, profound peace, and ecstasy, and they are given lessons by spirit entities. The last phase involves fatigue and fading visuals [22]. While participants are still able to speak and are aware of their environment during these experiences [37], the perception of time can be altered [73].

An ayahuasca experience resembles schizophrenic episodes; however, whether or not this herbal preparation or DMT are involved in a psychotic crisis is still a matter of debate. While some authors report that high levels of endogenous DMT are found in the urine and blood of individuals during a schizophrenic episode [147,148], others found no significant difference in DMT levels between schizophrenic patients and normal individuals [149]. In fact, it has been hypothesized that DMT is a homeostatic agent with psychotic suppression activity [4]. Additionally, and although having a resemblance, ayahuasca/DMT mainly produces visual hallucinations in healthy individuals, while auditory hallucinations are predominant in schizophrenia patients [56]. The few cases where psychotic episodes occurred were found to be transient in nature and resolved spontaneously [37,150]. Case-control and cross-sectional studies with experienced consumers of ayahuasca revealed that this hallucinogenic herbal concoction is safe, with rare adverse effects, and not associated with psychopathological nor neuropsychological deficits [37,43,151].

Short-term emotional distress can be a psychological consequence of DMT or ayahuasca use. Development of long-lasting psychosis is infrequent, mostly occurring in individuals after the concomitant use of other drugs, personal or family history of psychosis/non-psychotic bipolar disorders, as well as ongoing psychotic or maniac symptomatology [152]. In controlled clinical settings, factors that could predispose long-term psychological adverse effects are screened prior to administration of ayahuasca/DMT, their consumption being exceptionally safe in this scenario [152]. However, when ayahuasca is administered outside clinical settings or established ceremonial rituals, severe and unpredictable adverse psychological reactions that remain to be elucidated can be triggered [153].

Strassman et al. [65] evaluated neuroendocrine, cardiovascular, autonomic, and subjective effects following i.v. administration of 0.2 and 0.4 mg/kg of DMT to experienced hallucinogen users, mydriasis, elevated heart rate, blood pressure, and rectal temperature being observed, as well as higher blood concentrations of β-endorphin, corticotropin, cortisol, and prolactin, which are affected by serotonergic stimuli. Following oral ingestion by volunteers of ayahuasca (2 mL/kg; 0.5 mg/kg of DMT), Callaway et al. [13] also observed an increase in the growth hormone, prolactin and cortisol plasma concentrations, mydriasis, fluctuations in the respiration and heart rate, increased blood pressure, and oral temperature.

Tremor can be a physiological effect resulting from β*-*carbolines, which may be well derived from their interaction with serotonin binding receptors [154]. Harmine is considered an endogenous tremorigenic, higher levels being found in patients who suffer from essential tremor [155].

Santos et al. [72] evaluated the effects of ayahuasca on the lymphocyte subpopulation during a 24-h period. An increase in total lymphocyte percentages at 1.5 h, and a decrease at 4.5 h was recorded, although no significant differences compared to placebo at 24 h were seen. CD3 and CD4 lymphocytes suffered a transient decrease at 1.5 and 2 h, CD8 and CD9 lymphocytes were not found to be modified, and the levels of natural killer (NK) cells significantly increased at 1.5 and 2 h. It has been postulated that cytokine secretion and cell differentiation can be impacted by the peripheral activation of 5-HT_2A_ receptors on leukocytes by DMT [156,157]. As shown by House et al. [158], ayahuasca β-carboline alkaloids can also act on the immune system, a concentration-dependent suppression of IL-2 and IL-4 production, CD8 activity, B cell proliferation, and NK cell activity being observed following the exposure to harmaline.

## 10. Toxicological Effects

Significant adverse effects after consumption of classic psychedelics, mainly when used as pure drugs in controlled clinical studies, are rare [6,159,160]. However, when consumed in unsupervised settings, the judgement of individuals can be compromised [6], leading to the belief of having superpowers or flying ability [161], having actions out of the ordinary like jump out of buildings [162], or staring at the sun during long periods of time resulting in ocular damage [163,164].

The recreational use of psychedelics often results in what is called “bad trips” [1], characterised by symptoms like anxiety, palpitations, and visual distortions [165]. “Too little” DMT was also associated with unpleasant feelings, not allowing the consumer to achieve the desired development of the characteristic perceptual effects, only giving them a tensely dysphoric state [66]. However, it is sometimes difficult to assess the potential hazardous effects caused by the recreational consumption of DMT, since the drug is commonly used in combination with other illicit substances, such as psychostimulants, depressants, narcotics, cannabis, and alcohol [45]. By virtue of MAO inhibition, severe adverse effects can occur when ayahuasca or the β*-*carbolines alone are used concomitantly with selective 5-HT reuptake inhibitors (SSRIs) such as antidepressants [166], as this combination leads to accumulation of 5-HT at the synapses, resulting in a potentially fatal condition known as 5-HT syndrome. Other compounds that may have serotonergic effects (e.g., lithium and triptans through the activation of serotonin receptors, levodopa through the increase in serotonin release) can also precipitate this fatal condition and should be avoided with ayahuasca [167,168].

Vomiting and diarrhoea are often reported as adverse effects resulting from ayahuasca consumption [56], which may be due to increased central 5-HT stimulation of the vagus nerve and peripheral stimulation of the intestine [13]. However, in a study conducted by Sanches et al. [133] in depressive patients, the emetic effect was not considered as a cause of severe discomfort. Nausea and exhaustion are also commonly mentioned as side-effects. All of these effects are, however, considered to be transient, only persisting for one or two days, and easily manageable [169].

The treatment of tryptamine/serotonin intoxication is merely supportive and targeted at the symptoms. Activated charcoal can be beneficial when consumption is through the oral route; benzodiazepines to treat agitation, hypertension, and hallucinations; treatment with β-adrenergic antagonist when the patient has unstable vital signs [4].

Pic-Taylor et al. [170] investigated the toxicity of ayahuasca following *per os* administration to female Wistar rats. The lethal dose (corresponding to 15.1 mg/kg DMT) was found to be 50 times higher than the dose commonly used during religious ceremonies. Based on the application of behaviour tests (open field, elevated plus-maze, and forced swimming) on the same animal model, it was further suggested that, although increased serotonergic activation led to some neural degeneration, no permanent brain damage appears to occur [170]. Other rodent studies allowed to estimate that the LD_50_ values for humans are approximately of 1.6 mg/kg for DMT i.v. administration (a total dose of 112 mg for a typical 70 kg individual), and 8 mg/kg for DMT *per os* (a dose of 560 mg) [37], which is significantly higher than the average ceremonial dose of DMT (27 mg), giving DMT/ayahuasca a safety margin of approximately 20-fold. As such, a toxic dose of ayahuasca would consist of approximately 7.28 L for a 70 kg individual, which is unlikely to occur [22].

When consumed within the normal doses, solely serotonergic reactions are documented [37]. Excluding the cases of co-ingestion with other substances (e.g., consumption with 5-MeO-DMT) [171], there are no reports on deaths directly attributed to ayahuasca [22,172]. The only two cases of death reported in the literature that involved ayahuasca/DMT/harmala alkaloids were of a 71-year-old diabetic female who consumed *B. caapi* mixed with tobacco leaves (no information on quantities) [173] and a 25-year-old man who consumed herbal extracts containing harmala alkaloids and tryptamines (no information on quantities) [171], with no anatomical cause of death found in the autopsies. In the first case, blood analysis revealed only the presence of nicotine (710–1900 ng/mL) and the cause of death was determined as acute nicotine intoxication. In the second case, reported by Sklerov et al. [171], the following concentration ranges were obtained in the blood analysis: 0.01–0.02 mg/L for DMT, 0.04–0.07 mg/L for harmaline, 0.08–0.17 mg/L for harmine, 0.24–0.38 for THH and 1.20–1.88 mg/L for 5-MeO-DMT. The cause of death was undetermined. However, a few cases of suspected deaths involving ayahuasca consumption have been reported in the media.

In a recent study, Colaço et al. [174] submitted Wistar rats to a chronic 28-day treatment using the same ayahuasca samples as a previous referred study [170] at doses 2 times higher (corresponding to 4.28 mL/kg of ayahuasca, 0.52 mg/kg of DMT, 5.16 mg/kg of harmine, 0.342 mg/kg of harmaline, and 0.66 mg/kg of THH) than the common ritual dose of ayahuasca. Haematological analysis (i.e., haemoglobin, total haematocrit, erythrogram, leukogram, corpuscular volumes), and biochemical analysis for hepatic function (aspartate transaminase, alanine transaminase, and alkaline phosphatase), renal function (urea and serum creatine), and tissue damage (lactate dehydrogenase) were performed, with no reports on toxic effects [174]. Furthermore, a one-year study comparing regular ayahuasca users with controls [151] showed no indication that long-term ayahuasca use could induce psychologic maladjustment, mental health deterioration, or cognitive impairment. No decreased cognitive function nor increased mental health issues were associated with populations who have a life-time use of these mind-altering substances, particularly as part of religious ceremonies [175,176].

Case reports of intoxication with β-carbolines are related to the oral ingestion of *P. harmala* seeds at concentrations varying between 50–150 g [177,178,179] or unknown [138]. Harmaline, which constitutes 3% of the seeds and is present in low amounts in ayahuasca, is two times more toxic than harmine, inducing tremor, convulsion, respiratory paralysis, hypothermia, CNS depression, visual trouble, delirium, loss of coordination, paralysis, and sometimes hallucinations, when consumed in high doses [177]. Toxic effects usually appear 3–4 h after ingestion, with the first symptoms being nausea and vomiting, followed by an altered mental state and other neurological presentations.

As pregnant women also participate in ceremonial ayahuasca consumption, studies should be pursued in order to assess possible toxic effects that might occur during pregnancy [180]. Experimental data recorded in pregnant rats dealing with the in utero toxicity of ayahuasca alkaloids are contradictory, with some authors suggesting toxicity [181] while others do not [182]. Oliveira et al. [183] first reported the evidence of toxic effects in pregnant rats due to chronic ayahuasca consumption during gestational days 6–20, at doses 10 times higher (14 mL/kg of ayahuasca) than the normal human dose. These animals presented decreased food consumption and weight gain, accompanied with increased relative liver weight, an indication of hepatotoxicity. Effects at the fetal level included visceral and skeletal malformations, dilated lateral and third ventricles, and decreased body weight [183]. Nevertheless, such data appear to be of limited relevance and should be extrapolated to humans with caution since, from field observations, pregnant women attending the religious ceremonies tend to use ayahuasca less frequently, and in reduced quantity [180]. Although more investigation on this subject is needed, there are a few scientific evidences suggesting that no psychiatric nor neuropsychological problems are seen in adolescents who were exposed to ayahuasca before birth [180].

## 11. Substance Dependence and Tolerance

As a Schedule I controlled substance, DMT can be seen as an addictive substance, associated with substantial health risks [56]. Notwithstanding, studies have been contradicting this, as no compulsive drug-seeking precipitated by consumption of DMT or ayahuasca has been reported in humans [37]. Psychedelics, including DMT and ayahuasca, are in fact seen as safer substances than cocaine, opiates, or even the widely used nicotine and alcohol, with the advantage of lacking the abuse potential, characteristic of these former drugs [37]. As reviewed by Gable et al. [37], reports of abstinence syndrome after termination of DMT consumption are unknown.

Studies with repeated administration of DMT to volunteers have seen little or no drug tolerance [64,149]. Comparing long-term users and occasional consumers of ayahuasca, Bouso et al. [184] found that following ingestion of a single dose, both groups were associated with lower scores on working memory and performance improvement, but only the occasional users had an impaired performance in strategic planning. Thus, greater prior exposure to ayahuasca by long-term users was associated with drug-induced neuromodulatory or compensatory effects, resulting in reduced cognitive incapacitation. Further reports on tolerance solely included slight changes in the release of growth hormone, adrenocorticotropic hormone, and prolactin, which were found to be decreased following a second administration, and lower response in the systolic blood pressure and heart rate [65,185]. DMT did not elicit tolerance in animal models like squirrel monkeys [186] and cats [187]. Additionally, LSD is not capable to produce cross-tolerance to DMT [188], contrary to what happens with other classic hallucinogens like mescaline or psilocybin [189,190]. The absence of cross-tolerance suggests a distinguished pharmacodynamic behaviour for DMT, which makes it a quite unique substance among classic hallucinogens.

## 12. Potential Therapeutic Benefits

In the last 20 years, several pre-clinical and clinical studies have revealed the therapeutic properties of psychedelics [6,191]. Among the psychedelic-rich traditional medicines, ayahuasca may be the better-known [192] due to its use in the treatment of several neurological disorders like depression, anxiety, and substance abuse [193,194,195].

Changes in cortical thickness of midline brain structures, such as decrease in the posterior cingulate cortex and increase in the anterior cingulate cortex, were detected in a magnetic resonance imaging study after long-term use of ayahuasca [71,112,196]. The authors postulated that the preservation of ayahuasca users’ neuropsychological function could be explained by these structural differences [112]. DMT can also induce structural and functional plasticity in prefrontal cortical neurons, namely increased dendritic spine density and frequency/amplitude of spontaneous excitatory postsynaptic currents [197], explaining both DMT and ayahuasca anxiolytic and antidepressant effects. In long-term ayahuasca users (>10 years), ratings of hopelessness were reduced [19]; a marked improvement in depressive symptoms for up to 21 days was also observed after a single dose [115], further suggesting its potential clinical utility. As part of the Hoasca Project, Grob et al. [198] found that in comparison with matched controls, ayahuasca users from a syncretic church had a remission in all previous alcohol, depressive, and anxiety disorders. Marked improvement in confidence, optimism and emotional maturity has been also observed, ayahuasca users also being more energetic, persistent, and reflective [199]. In this context, it is however difficult to separate the actual positive effects of the substances *per se* from those related with social factors, like the inclusion in a strong religious group [22]. As such, this must be borne in mind in the interpretation of these, and similar results. Bouso et al. [112] found that ayahuasca consumers scored significantly better on “harm avoidance” in comparison with matching controls, as well as on other variables dealing with working memory, executive function, set shifting, and personality. The same group compared 127 regular ayahuasca users with 115 actively religious non-user controls [151] over one year, aiming to assess the possible effects of regular ayahuasca use on general psychologic well-being, mental health, and cognition. Regular consumers scored higher on psychosocial well-being and had better resistance to emotional interference, as well as on working memory. A study covering adolescent consumers of ayahuasca in a religious context found slight differences in terms of psychopathological profile when compared with non-consumers [200]. The ayahuasca group showed less anxiety symptoms, less concerns over body image, and better capacity of attention. Another study aiming to evaluate neuropsychological performance found no overall differences between ayahuasca and non-ayahuasca groups of adolescents [201]. Chronic consumption of psychedelics was suggested to alleviate mental illness distress, reduce suicide attempts and suicidal planning/thinking, while the use of non-psychedelic substances has been considered a suicide risk factor [202]. Ayahuasca users had seen positive effects on their mental health, including improved psychological well-being, decreased alcohol consumption, reduced impulsivity, boosted mood, and improved cognitive function [203]. Patients diagnosed with treatment-refractory depression have been found to benefit from the consumption of a single dose of ayahuasca [115,133,194]. This offers a potential and exciting new alternative for treating these diseases, especially as lack of effectiveness and late onset of therapeutic activity are often associated with the currently available antidepressants [56,197].

DMT also seems to induce potent anti-inflammatory effects through the binding and activation of the sigma-1 receptor, which might be relevant in neuropsychiatric diseases with a neuroinflammatory background. These consist, on one hand, of the inhibition of pro-inflammatory cytokines (IL-1β, IL-6, TNFα) production, and on the other of the enhanced release of anti-inflammatory cytokine IL-10 [204]. A role for DMT in the treatment of neurodegenerative disorders (e.g., Parkinson’s disease) may also be speculated due to the reported anti-inflammatory ability [205].

In spite of its specific role in the overall medicinal properties of ayahuasca being still unknown, harmine also appears to exert pharmacological properties that might be translated into new clinical drugs [56]. In fact, harmine and other harmala alkaloids that inhibit MAO have been long used as antidepressants in humans outside clinical practice, proving to modulate mood and anxiety [206]. Interestingly, harmine has been recently proposed to have additional important pharmacological roles, like the inhibition of angiogenesis and tumour growth due to the activation of the tumour suppressor p53 in endothelial cells [207], as well as antioxidant effects, through the increase in superoxide dismutase and catalase activity, and antidepressive properties [208].

## 13. Ayahuasca Metabolomic Aspects

Metabolomic studies on ayahuasca are extremely scarce. Very recently, the neuroprotective role of ayahuasca was investigated in human neuroblastoma cells (SH-SY5Y) challenged with 6-hydroxydopamine (6-OHDA), this being a suitable model to study the neuroprotective effect of substances on Parkinson’s disease (PD) [209]. SH-SY5Y cells were treated with samples of ayahuasca, fractions of its source plants (*B. caapi* or *P. viridis*), or individual alkaloid extracts (DMT or harmine) every 24 h, for 48 or 72 h. Using the 3-[4,5-dimethylthiazol-2-yl]-2,5-diphenyl tetrazolium bromide (MTT) assay to measure cell viability followed by an untargeted metabolomic approach that used multivariate statistical analysis (MSA) to treat metabolome data obtained with a ultra-performance liquid chromatography coupled to electrospray ionisation and time-of-flight (UPLC-ESI-TOF), the authors searched for compounds that could be correlated with the neuroprotective effects of ayahuasca, allowing the identification of promising drugs for PD treatment. The ayahuasca decoction, alcoholic extracts of its source plants, and the alkaloid extracts of harmine showed neuroprotective activity, and led to improved cell viability and neuronal cell proliferation. Both harmine and THH as well as other β-carbolines, exhibiting neuroprotective activity at the second time-point (72 h), were considered by the authors to be promising new drugs to be used for the development of PD treatments. However, through the untargeted metabolomic approach, other compounds present in ayahuasca source plants were correlated with the best neuroprotective profile at both time-points (48 and 72 h). Those included possible polyphenolic components, inositol, mannopyranosyl and galactopyranosyl derivatives, as well as unknown compounds defined as “possibly novel”, which need to be further isolated and identified. Using traditional phytochemical methods, these compounds would be difficult to detect since they are mostly minor metabolites being possibly present in the hydroalcoholic fractions, highlighting the benefit of using untargeted metabolomic analysis for drug discovery optimization.

## 14. Toxicological Analysis and Forensic Relevance

The herbal drug ayahuasca has been brought into the spotlight not only due to its use in religious rituals and clinical research, but also due to the increasing and widespread consumption in recreational contexts. In fact, such abuse has been hampering research progress and clinical application of hallucinogens for many decades. As such, comprehensive toxicological analysis of both biological samples and ayahuasca itself are important topics in a forensic context [210].

In addition to plasma, blood, and urine, the conventional biological matrices for the determination of DMT and β-carbolines [21,27,28,80,89,211,212], hair and sweat have been also considered for the detection of ayahuasca compounds [213,214]. The latter biological matrices show some advantages, mainly due to their easier and non-invasive obtaining, having already demonstrated several applications in forensics.

Urine drug tests with commercially available enzyme multiplied immunoassay techniques are commonly considered, amongst other tests [215]. By virtue of the short DMT half-life, this substance is considered a poor target for urine toxicology screening, often challenging confirmation of acute overdoses due to the false negative results [211]. Adding to this, a false positive result for amphetamine can be an outcome of these screening tests, due to the cross-reaction of DMT with sympathomimetic agents [215]. Liu et al. [216] presented two cases of patients who developed DMT intoxication, which required the identification of DMT and harmaline in biological samples by liquid chromatography coupled to tandem mass spectrometry (LC/MS/MS), in order to avoid a misdiagnosed amphetamine poisoning by urine screening tests. In this line, specific analytical methods that have proven suitable for determination and quantification of DMT and also other ayahuasca alkaloids in biological matrices, include high-performance liquid chromatography (HPLC) coupled to a fluorescence detector [21,28], gas chromatography with nitrogen-phosphorus detection [21,28], LC/MS/MS [27,89,212], and gas chromatography coupled to mass spectrometry (GC/MS) [213], whose validation parameters can be seen in Table 4.

Non-targeted urine toxicology screen of the most abundant harmala alkaloids found in ayahuasca beverages and in human studies, namely harmine, harmaline, and THH, has been proposed as an alternative approach to confirm ayahuasca intoxication. The first report on a real case of harmala alkaloids detection allowing the identification of an ayahuasca intoxication, using UHPLC-QToF as a non-targeted urine drug screening method, was published by Pope et al. [211]. In this case, a 40-year-old male, with previous medical history of polysubstance abuse, entered the hospital after drinking alcohol and ayahuasca, with signs of persecutory delusions, and in a threatening state (both verbally and physically). A drug screen for hallucinogens came negative, although clinical information strongly pointed to ayahuasca use. Therefore, an alternative approach to identify ayahuasca consumption was sought, the detection data on harmala alkaloids being included in the toxicology screening library. Both harmaline and THH were found in the patient’s urine, allowing confirmation of ayahuasca consumption [211].

In addition, Lo Faro et al. [210] recently covered the analytical techniques commonly used for the identification and quantification of ayahuasca alkaloids in confiscated materials. Analytical tools include hyphenated HPLC and GC systems, [49,217], namely nitrogen-phosphorus detector or MS [218,219,220]. A complete, rapid, simple, and reliable analytical method based on UHPLC-MS/MS has been recently validated for the detection of DMT and harmala alkaloids in botanical samples [221]. Through its application in real-seized powder samples, only DMT was detected at concentrations ranging from 31.5 to 46.5 mg/g. This allowed to conclude that these DMT powder samples were either for use in conjunction with MAO inhibitor drugs *per os*, or their consumption was carried out by smoking or inhalation, which can result in a potentiation of the occurrence of intoxication episodes due to the increased bioavailability of DMT [49,221].

Longo and Rabi described a method capable of determining the handling of ayahuasca through the analysis of users’ fingerprints by matrix-assisted laser desorption ionization associated with mass spectrometry imaging (MALDI-MSI) [222]. This technique used the harmala alkaloids harmine and harmaline, plus DMT, as biomarkers of ayahuasca exposure. The establishment of a connection between a specific individual and its contact with ayahuasca, fundamental to law enforcement for the identification of cases of abuse, is a challenge, and therefore the development of these methods is essential in the context of a forensic investigation.

Sweat samples were collected during and after ayahuasca intake from 21 members of a syncretic religious ceremony, by using patches attached to the volunteer’s skin; the average concentrations per patch were 73.02 ng DMT, 903.40 ng harmaline, and 95.13 ng harmine [213]. Nevertheless, no analytes were detected in 14.3% of the samples, and only 52.4% samples presented positive results for all three analytes. The lack of analyte detection could be associated with low sweat production/collection of some individuals.

Drug concentrations were determined by LC-MS in post-mortem samples of a fatal intoxication of a 25-year-old male involving the oral consumption of an ayahuasca preparation followed by ingestion of tryptamines 4 h later. [171]. Biological specimens involved heart and peripheral blood, gastric content, bile, brain, kidney, liver, and urine. Due to the rapid metabolisation and excretion, DMT was not detected in the brain, kidney, and liver, unlike 5-MeO-DMT and the other ayahuasca alkaloids, which were detected in all biological matrices. Concentrations of DMT were 0.02 mg/L in heart blood, 0.01 mg/L in peripheral blood, 3.3 mg/L in gastric samples, 0.57 mg/L in bile, and 0.89 mg/L in urine. The concentrations of 5-MeO-DMT were higher in all the samples where DMT was present: 1.88 mg/L in heart blood, 1.20 mg/L in peripheral blood, 201.6 mg/L in gastric fluid, 9.81 mg/L in bile, and 9.59 mg/L in urine, which explained the fatal intoxication. Considering the post-mortem concentrations of the harmala alkaloids, with exception of the gastric content, where the amounts of harmaline were 10-fold greater than the amounts of THH, THH presented higher concentrations in all the biological samples: 0.38 mg/L in heart blood, 0.24 mg/L in peripheral blood, 4.78 in bile, 0.43 mg/L in brain, 6.89 mg/L in kidney, and 13.24 mg/L in liver. The amounts of THH in all specimens were also higher than that of DMT.

Several factors can influence the concentrations of these alkaloids in biological samples, namely the varying amounts of ayahuasca being ingested, interindividual metabolic variations, since slow and rapid metabolisers of harmine due to CYP2D6 polymorphisms lead to interindividual changes in its biotransformation [223], and also existing variations in the composition of ayahuasca preparations.

In forensic investigations, it is important to determine the alkaloid content of the concoctions, allowing the application of suitable protocols for overdose investigation. Taxonomic identification of the botanical species used in the preparation of ayahuasca is also important in tracking the source of the product and giving information about the vendor/manufacturer [224]. Identification of an ayahuasca preparation is manly performed by the confirmation of DMT in samples of the herbal preparation or botanical samples, which is however insufficient to identify the specific psychoactive plant species. Lesiak and Musah [224] presented a method to accomplish this identification, based on the unique chemical fingerprint of the ayahuasca preparations, using chemometric processing of direct analysis in real time-high resolution mass spectrometry (DART-HRMS). Readers are referred to the review by Gaujac et al. [225] and references therein on analytical techniques used towards the detection and quantification of tryptamines and harmala alkaloids in plant sources and ayahuasca beverages.

## 15. Conclusions and Future Perspectives

**Figure 6:**
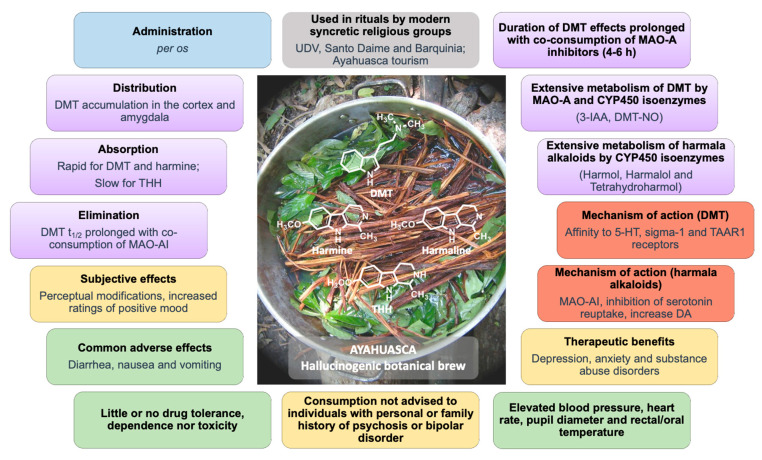
Pharmacokinetic and pharmacodynamic aspects of ayahuasca. 3-IAA: 3-Indole-acetic acid; 5-HT: Serotonin; DA: Dopamine; DMT: *N*,*N*-Dimethyltryptamine; DMT-NO: DMT-*N*-oxide; MAO: Monoamine oxidase; TAAR1: Trace amine-associated receptor type 1; THH: Tetrahydroharmine, UDV: *União do Vegetal*.

Plant-derived hallucinogens are probably the oldest drugs consumed by mankind. As herein reviewed, ayahuasca is a botanical concoction leading to profound psychoactive effects due to complex pharmacological and pharmacokinetic interactions between active alkaloids known to occur in the leaves of *P. viridis* and the bark of *B. caapi*, but also in other plants that are commonly used in the preparation. In this work, a comprehensive review on the toxicokinetics and toxicodynamics, clinical, and forensic impact of ayahuasca’s main active alkaloids was attempted (Figure 6).

Upon intake, DMT suffers a rapid and extensive first-pass metabolism mediated by MAO-A enzymes; being orally inactive, it cannot reach systemic circulation, nor the brain. However, when consumed with β-carbolines, which are potent transient inhibitors of MAO enzymes, DMT metabolism is shifted to a less-active metabolic pathway; thus, sufficient amounts of unmetabolised compound can reach the CNS and exert the desired effects. DMT has affinity for a variety of serotonergic and non-serotonergic receptors, namely 5-HT_1A/1B/1D/2A/2B/2C/6/7_, sigma-1, and TAAR1, which could explain the huge variety of potential therapeutic effects reported for ayahuasca. The main target of DMT is, however, the 5-HT_2A_ receptor; this interaction is the main event underlying the hallucinogenic properties of DMT and ayahuasca.

Both adverse effects of ayahuasca as well as its possible applications in the clinical area have recently been the subject of high research interest by the scientific community. However, there has been an increase in the recreational consumption of DMT and ayahuasca, leading to a higher misuse and development of unpredictable health hazards. The development of analytical methods for the determination of alkaloids in biological and botanical samples is thus of utmost relevance in clinical and forensic toxicology.

Almost all available studies involving the therapeutic research of ayahuasca, DMT, and other psychedelics evoke a series of promising beneficial outputs of their use. Both ayahuasca and its alkaloids have shown potential for the treatment of depression, anxiety, as well as substance-abuse disorders, both in preclinical and observational studies [226]. However, there is still a need for more extensive clinical research on the use of these substances [6,227]. Ayahuasca consumption is reported as being safe in healthy individuals, only provoking some common unpleasant and transient adverse effects—vomiting, diarrhoea, nausea, and exhaustion. Serious adverse and toxic effects, potentially leading to death, have only been reported for individuals with concomitant use of other drugs, and with personal or family history of psychiatric disorders. Both ayahuasca and DMT consumption do not lead to addiction, and little or no tolerance has been reported after repeated consumption. All these proven benefits and lack of serious health effects are not consistent with the highly restricted legal status of DMT.

The interest in the use of psychedelics in clinical studies has seen a recent upsurge, leading to the development of appropriate procedures addressed to minimise possible adverse reactions, thus maximizing safety. The detailed guidelines proposed by Johnson et al. [228], concerning the administration of hallucinogens to humans in clinical trials, encompass the following: (1) a methodologic selection of volunteers, who must be in good general health; (2) the presence of at least two staff members (“monitors”) who should have human relation skills, knowledge of the altered states of consciousness, and be well-informed about medical and psychological markers related to adverse reactions; (3) the importance of an aesthetically pleasing and comfortable environment; (4) a well preparation of volunteers, including review of the consent form, with the full description of study procedures, the possible range of experiences and adverse effects; (5) building trust between volunteers and monitors through a series of meetings; (6) availability of a physician during hallucinogens administration sessions, and appropriate medication; and (7) post-session meetings between the primary monitor and volunteers for subsequent safety check. If these criteria are met, DMT and ayahuasca can be used with a very safe profile for the treatment of a range of psychiatric and medical conditions.

## Figures and Tables

**Table 1:** Chemical composition and alkaloid content of ayahuasca preparations.

Ayahuasca Preparations	DMT (mg/mL)	Harmine (mg/mL)	Harmaline (mg/mL)	THH (mg/mL)	Total Alkaloids (mg/mL)	Reference
Rio Purús ^1^	0.13	0.15	n.a.	0.05	0.33	[26]
UDV	0.24	1.70	0.20	1.07	3.21	[13]
Pucallpa ^2^	0.60	4.67	0.41	1.60	7.28	[16]

^1^ A river that flows through the countries of Brazil and Peru. ^2^ A city from Peru. DMT: *N*,*N*-Dimethyltryptamine; THH: Tetrahydroharmine; UDV: *União do Vegetal*; n.a.: Information not available.

**Table 2:** Physicochemical properties of *N*,*N*-dimethyltryptamine (DMT), harmine, harmaline, and tetrahydroharmine (THH).

Ayahuasca Alkaloids	LogP	MW (g/mol)	Mp (°C)	pK_a_
DMT	2.573	188.27	44.6–46.8	8.68
Harmine	3.56	212.25	261	7.6
Harmaline	2.1	214.3	249–250	n.a.
THH	1.9	216.28	n.a.	n.a.

Mp: Melting point; MW: Molecular weight; n.a.: Information not available.

**Table 3:** Pharmacokinetics of *N*,*N*-dimethyltryptamine (DMT), harmine, harmaline, and tetrahydroharmine (THH), when administered alone or in ayahuasca preparations.

Ayahuasca Alkaloids	Route of Administration, Doses and Onset/Duration of Effects		Absorption		Distribution		Excretion	
	Individual	Ayahuasca	Individual	Ayahuasca	Individual	Ayahuasca	Individual	Ayahuasca
DMT	Smoked (40–50 mg): onset of effects at 1–15 s, peak at 5 min, and duration of 1 h; Injected: similar effects	Oral: peak of effects at 1.5–2 h; duration of effects 4–6 h	Extensive first pass metabolism (no bioavailability)	T_max_^1^ = 1.5–1.8 h	Rapid distribution through the liver and kidney (5–10 min), and brain (10–15 min)	-	t_1/2_^2^ = 5.7 min for brain, 9.6 min for liver, 17.2 min for kidney, and 15.8 min for blood Oral: 3-IAA (97%), DMT-NO (3%) Smoked: 3-IAA (63%), DMT-NO (28%), DMT (10%)	t_1/2_^2^ = 1.07–4 h Recovery in urine: 3-IAA (50%), DMT-NO (10%), DMT (1%), 2-MTHBC and NMT (0.2%)
Harmine	Oral (20–50 mg): onset of effects at 20–30 min, peak at 30 min–1 h, and duration of 6–8 h; i.m. injection: onset of effects at 5–10 min, peak at 30 min, and duration of 3–5 h	Oral	T_max_^1^ = 0.56–2.7 h Bioavailability: 17.11 %	T_max_^1^ = 1.7 h	High concentrations in the liver, kidney, spleen, and lung	-	-	t_1/2_^2^ = undetectable-2 h
Harmaline	-	Oral	T_max_^1^ = 0.73–4 h Bioavailability: 1.09 %	T_max_^1^ = 2.4 h	High concentrations in the liver, kidney, spleen, and lung Found in the brain	-	-	t_1/2_^2^ = 1.95–2.1 h
THH	-	Oral	-	T_max_^1^ = 2.5–3 h	-	-	-	t_1/2_^2^ = 4.68–8.9 h

^1^ Time required to obtain the maximum plasma concentration. ^2^ In vivo half-life of elimination. 2-MTHBC: 2-Methyl-1,2,3,4-tetrahydro-β-carboline; 3-IAA: 3-Indole-acetic acid; DMT-NO: DMT-*N*-oxide; NMT: *N*-Methyltryptamine; (-): Not mentioned.

**Table 4:** Analytical methods for detection and quantification of the major compounds of ayahuasca in biological samples.

Analytes	Analytical Method	Matrix	Recovery (%)	Linearity (Mean R^2^)	LOD and LOQ	Reference
DMT; harmine, harmaline, THH, harmol and harmalol	GC-NPD; HPLC-FLD	Plasma	74 (DMT); >87 (harmine, harmaline, THH, harmol and harmalol)	0.9946 (DMT); >0.9916 (harmine, harmaline, THH, harmol and harmalol)	0.5 ng/mL and 1.6 ng/mL for DMT; 0.1 ng/mL and 0.5 ng/mL for harmine; 0.1 ng/mL and 0.3 ng/mL for harmaline, harmol and harmalol; 0.3 ng/mL and 1.0 ng/mL for THH	[21]
DMT; harmine, harmaline, and THH	GC-NPD; HPLC-FLD	Plasma	Not available	0.994 (DMT)	0.5 ng/mL and 5 ng/mL for DMT; 0.1 ng/mL and 2.0 ng/mL for harmine; 0.05 ng/mL and 1.0 ng/mL for harmaline; 0.1 ng/mL and 1.9 ng/mL for THH	[28]
DMT, harmine, harmaline, THH, harmol, harmalol, and other metabolites	LC-MS/MS (ESI)	Urine	Not available	0.9997 (DMT), 0.9996 (harmine), 0.9992 (harmaline), 0.9990 (THH), 0.9995 (harmol), 0.9986 (harmalol), 0.9995 (NMT), 0.9991 (2-MTHBC), 0.9995 (DMT-NO)	0.12 ng/mL and 5 ng/mL for DMT; 0.18 ng/mL and 5 ng/mL for harmine; 0.07 ng/mL and 5 ng/mL for harmaline; 0.21 ng/mL and 5 ng/mL for THH; 0.57 ng/mL and 5 ng/mL for harmol; 0.18 ng/mL and 5 ng/mL for harmalol; 0.04 ng/mL and 5 ng/mL for NMT; 0.14 ng/mL and 5 ng/mL for 2-MTHBC; 0.07 ng/mL and 5 ng/mL for DMT-NO	[27]
DMT, harmine, harmaline, THH, harmol, harmalol, and other metabolites	LC-MS/MS (HESI)	Blood	60.28–76.31	0.9995 (DMT), 0.9990 (harmine), 0.9986 (harmaline), 0.9992 (THH), 0.9992 (harmol), 0.9994 (harmalol), 0.9993 (NMT), 0.9986 (2-MTHBC), 0.9990 (DMT-NO)	0.45 ng/mL and 1 ng/mL for DMT; 0.25 ng/mL and 1 ng/mL for harmine; 0.22 ng/mL and 1 ng/mL for harmaline; 0.36 ng/mL and 1 ng/mL for THH; 0.30 ng/mL and 1 ng/mL for harmol; 0.38 ng/mL and 1 ng/mL for harmalol; 0.32 ng/mL and 1 ng/mL for NMT; 0.33 ng/mL and 1 ng/mL for 2-MTHBC; 0.25 ng/mL and 1 ng/mL for DMT-NO	[89]
DMT, harmine, harmaline, and THH	LC-MS/MS (HESI)	Plasma	89.4–107.7	0.9984 (DMT), 0.9934 (harmine), 0.9972 (harmaline), 0.9908 (THH)	0.1 ng/mL and 0.2 ng/mL for DMT; 0.1 ng/mL and 0.3 ng/mL for harmine; 0.1 ng/mL and 0.4 ng/mL for harmaline; 0.1 ng/mL and 0.4 ng/mL for THH	[212]
DMT, harmine, and harmaline	GC-MS	Sweat	72.1–90.1	0.9922 (DMT), 0.9943 (harmine), 0.9931 (harmaline)	10 ng/patch and 20 ng/patch for DMT; 15 ng/ patch and 20 ng/patch for harmine; 15 ng/ patch and 20 ng/patch for harmaline	[213]

DMT: *N*,*N*-Dimethyltryptamine; ESI: Electrospray ionization; GC-MS: Gas chromatography coupled to mass spectrometry; HESI: Heated electrospray ionization; HPLC-FLD: High-performance liquid chromatography coupled to a nitrogen-phosphorous detector; LC-MS/MS: Liquid chromatography coupled to tandem mass spectrometry; LOD: Limit of detection; LOQ: Limit of quantification; R^2^: Coefficient of determination; THH: Tetrahydroharmine.
